# Common characteristics of open source software development and applicability for drug discovery: a systematic review

**DOI:** 10.1186/1478-4505-9-36

**Published:** 2011-09-28

**Authors:** Christine Årdal, Annette Alstadsæter, John-Arne Røttingen

**Affiliations:** 1 The Norwegian Knowledge Centre for the Health Services, P.O.Box 7004, St. Olavs plass, N-0130 Oslo, Norway; 2 University of Oslo, Department of Health Management and Health Economics, P.B. 1089 Blindern, N-0317 Oslo, Norway

**Keywords:** open source, drug discovery, pharmaceutical innovation, neglected diseases

## Abstract

**Background:**

Innovation through an open source model has proven to be successful for software development. This success has led many to speculate if open source can be applied to other industries with similar success. We attempt to provide an understanding of open source software development characteristics for researchers, business leaders and government officials who may be interested in utilizing open source innovation in other contexts and with an emphasis on drug discovery.

**Methods:**

A systematic review was performed by searching relevant, multidisciplinary databases to extract empirical research regarding the common characteristics and barriers of initiating and maintaining an open source software development project.

**Results:**

Common characteristics to open source software development pertinent to open source drug discovery were extracted. The characteristics were then grouped into the areas of participant attraction, management of volunteers, control mechanisms, legal framework and physical constraints. Lastly, their applicability to drug discovery was examined.

**Conclusions:**

We believe that the open source model is viable for drug discovery, although it is unlikely that it will exactly follow the form used in software development. Hybrids will likely develop that suit the unique characteristics of drug discovery. We suggest potential motivations for organizations to join an open source drug discovery project. We also examine specific differences between software and medicines, specifically how the need for laboratories and physical goods will impact the model as well as the effect of patents.

## Background

Innovation through an open source model has proven to be successful for software development. Well-known examples such as the Linux operating system and Apache web server have demonstrated that open source methods can create market leaders [1,2].

This success has led many to speculate if open source can be applied to other industries with similar success. The ingredients of open source generally deemed attractive for transfer are the collaborative nature of development and the open access to the intellectual property. Pharmaceuticals are an often mentioned example for possible transfer and adaptation. The World Health Organization's Consultative Expert Working Group for Research and Development Financing and Coordination has been requested to evaluate open source drug discovery. There are also several open source drug discovery projects already underway. The Synaptic Leap hosts a project to develop a new synthesis of the schistosomiasis drug, *praziquantel*, and CSIR Team India Consortium hosts a project identifying new targets for tuberculosis. These projects attempt to link up like-minded scientists globally to develop new drugs quickly without high, patent-protected prices, making medicines more accessible.

This is a simplistic and ideal description of a potential utilization of open source. To discuss the applicability of open source to other contexts seriously, we need to understand more about the phenomenon. This paper attempts to analyze the existing, empirical research regarding open source software development and single out those characteristics that are important when designing and building new open source models. We attempt to present the evidence in such a way that it is useful for researchers, business leaders or government officials who may be interested in applying the concepts of open source to novel areas. We apply our findings specifically to drug discovery.

We have chosen a multidisciplinary and mixed-methods systematic review to present the research. A multidisciplinary approach allows for the examination of a wide range of research evaluated from multiple perspectives - economic, legal, software engineering, etc. A systematic review is a method of evaluating large bodies of evidence in a systematic, transparent and reproducible manner [3]. The aim is to give an unbiased reproduction of the current evidence addressing the research question, what are the common characteristics and barriers of open source software development.

### The myriad of "open" concepts

Firstly, it is important to define what we mean by "open source" as there is a myriad of "open" concepts with considerable overlap. The Open Source Initiative has removed much of the ambiguity with "open source" as pertaining to software with their 10-point Open Source Definition [4], a detailed definition giving ten criteria that a license must comply with in order to be recognized as open source. The major components are:

• Access to the source code: The lines of code that comprise the source code are the instructions running the software. If an individual wants to make changes to a software program, he/she generally needs access to the source code.

• Free redistribution: An individual may use all or parts of the open source software as a component in a larger software application without the requirement of a royalty or a fee.

• Creation of derived works: Individuals are allowed to change or expand the open source software and distribute the newly created software.

The license defines the formal definition of open source as it relates to the management of the intellectual property (which is typically copyright). However, the concept of open source also conveys a collaborative approach to innovation. Programmers work together virtually to develop a software program. They are not employed by a single organization. They typically volunteer for tasks and come and go from a project at will.

A concept closely related to open source is "free software". It is fundamentally the same as open source but with a political twist - adherents to free software believe that all software should be made freely available and that proprietary software should not exist. The Open Source Community takes a more flexible approach, allowing proprietary software to use open source components so long as the license allows for it. Sometimes the two terms are combined in FLOSS (free/libre/open source software).

"Open innovation" conveys a much broader idea than open source. University of California at Berkeley's Henry Chesbrough has championed open innovation, which encourages companies to actively supplement their internal knowledge stocks with external sources [5]. Instead of relying solely on internal research, companies that follow open innovation business models actively purchase or license ideas from external organizations and/or look to the public domain for possible business models. Unlike open source, open innovation may involve contracting with the intellectual property rights holder and paying a royalty.

"Open access" is a general term with varying meanings depending upon the context. When used to discuss content, it generally means the free access to books, journals, media, etc. [6]. This allows individuals to read, copy, print or distribute the materials free-of-charge. Unlike open source, it does not allow individuals to modify the materials without the author's consent.

"Open knowledge" takes the concept of open source and generalizes it beyond computer software. It is intended to cover copyrighted data (such as music, books, scientific data, etc.) but not software as this is adequately addressed by open source. It is defined in an 11-point definition by the Open Definition [7] as knowledge that anyone is "free to use, reuse, and redistribute -- subject only, at most, to the requirement to attribute and share-alike."

This review focuses solely on open source software development because it is this concept that has become the model for the other "open" concepts. Being the original model, it is the oldest, most established and the most studied. Articles regarding open source drug discovery are not included in the systematic review but are included in the Discussion section.

### Open source software development research themes

The existing research on open source software development is varied and plentiful. Major themes of the research include the analysis of developers' and firms' motivations, license choice, successful implementations and the impact on innovation. Von Krogh et al. [8] have analyzed the existing research on open source software developers' motivations and grouped the research literature into two main phases. The early research phase examined why developers contribute. The subsequent phase examined the relationships between developers' motivations, contributions and institutional arrangements.

Researchers have also analyzed the applicability of open source in other domains such as drug discovery. Maurer and Scotchmer reviewed the empirical research of open source software development and "provide a snapshot of the emerging open source phenomenon" [9]. They examine the incentives, organization, knowledge gaps and its potential as a model in drug discovery and geographic information systems.

Several other researchers examine applicability to other domains theoretically. Müller-Seitz (2009) examines the parallels and differences between open source software development and open biotechnology using Cambia's BiOS as a case example [10].

Our paper builds on Maurer and Scotchmer's work in that both examine characteristics of open source software development. Where as Maurer and Scotchmer comprehensively detail open source software characteristics, we have done a systematic review and only focus on those characteristics that we believe are applicable for drug discovery. We then take these characteristics one step further and discuss transferability.

### Brief description of open source software development

Open source has origins to the beginning of computer software development. Although not called open source at the time, early software was shared freely amongst developers and not considered a commercial product [11].

In the 1970's to 80's the commercial potential of computer software became evident. Microsoft entered the operating system business in 1980 [12]. AT&T began selling a licensed version of Unix in 1982 [13]. Richard Stallman, a programmer at MIT's artificial intelligence laboratory, became alarmed by the increasing commercialization of computer software. In reaction he launched the GNU Project (a recursive acronym for GNU's Not Unix) in 1983, creating an open source Unix-like operating system. In 1985 he launched the Free Software Foundation whose aim is to promote "free software" including the political twist mentioned earlier. All free software originally was licensed under a new type of license called "copyleft", named to emphasize the difference from the copyright [14]. GNU General Public License (GPL) was the first example of a copyleft license, giving anyone the freedom to use, modify and distribute software with the caveat that all modifications must also adhere to the GPL. Hence, all future products became "infected" by the original license and must remain GPL-compliant (free to use, modify and distribute). This caveat earned copyleft licenses the designation "viral licenses" [15].

The most successful and famous example of free and open source software is Linux, a Unix-like operating system. It was started by Linus Torvalds in 1991 and uses a GPL license [11]. Apache web server started in 1995. Apache does not use a GPL license; they have created a non-viral equivalent that abides by the Open Source Definition [11]. Individuals or firms can commercialize Apache software or combinations of Apache and proprietary software. IBM became actively involved in Apache development in 1998, giving the open source movement significant commercial credibility [11]. Smaller, less well-known open source projects are often hosted on SourceForge.net, the largest open source software developer website. It hosts more than 260,000 open source software projects and has more than 2.7 million registered users as of March 2011 [16].

## Methods

We have reviewed the existing empirical literature pertaining to our research questions:

• What are the common characteristics for initiating and maintaining an open source software development project?

• What are the barriers for developing an open source software development project?

We have carried out this secondary research in a transparent and reproducible manner by performing a systematic review. A systematic review follows a prescribed path summarizing large bodies of evidence in accordance with scientific strategies [3]. Bias and error are reduced because the researcher must follow a pre-defined search strategy, including target databases, search words and explicit inclusion and exclusion criteria. The researcher should not randomly include other studies not following these criteria because they may bias the results of the systematic review. The search is performed at one period of time and may be updated annually or bi-annually to incorporate any new research. Where as it may appear unorthodox to limit the research findings in this way, the results should be an unbiased and objective account of the existing research as long as the researcher has appropriately defined the review criteria. To our knowledge no other systematic reviews pertaining to open source software development exist.

### Search strategy and study selection

**Figure 1:**
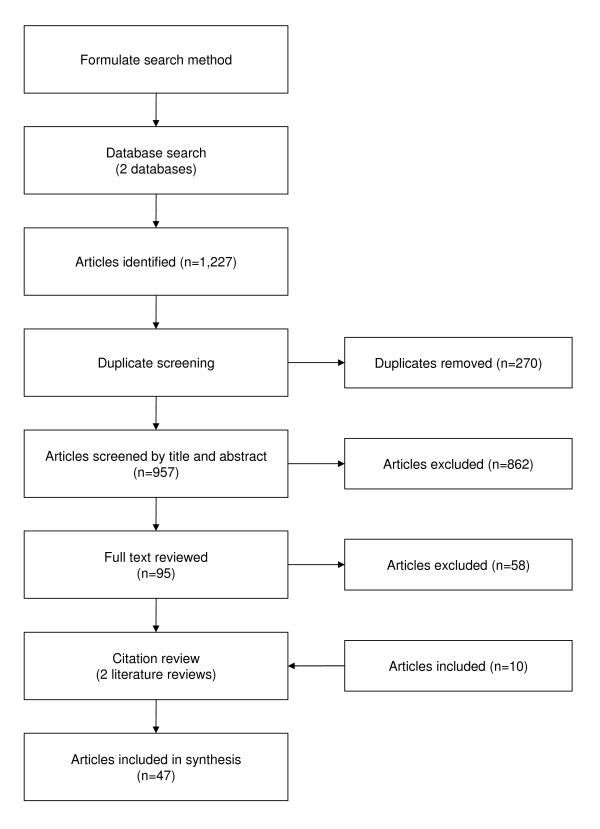
**Article selection**. ISI Web of Knowledge and Google Scholar were both searched with the key phrase "open source" in the title in addition to a number of keywords. These searches returned 1,227 articles which were screened for relevance according to the inclusion criteria. In the end 47 articles were included in the synthesis.

Figure 1 maps the process by which articles were selected for the systematic review. We wanted to ensure that the search would retrieve articles from a variety of disciplines such as economics, law, information systems, management and social sciences. Trial searches performed in discipline-specific academic databases returned few articles of value. Therefore, we decided to use multidisciplinary and comprehensive databases, eventually choosing ISI Web of Knowledge and Google Scholar.

Since it is not possible to search Google Scholar by topic (or abstract), the keywords were expanded to include: innovation(s), lesson(s), developments, understanding and determinants. The search retrieved all articles where at least one of the keywords in addition to the phrase "open source" was found in the title. It was necessary to restrict the search to words found in the title in order to constrain the search results to a manageable level (less than 10,000 results). The search was also constrained to the subject areas of:

• Biology, life sciences and environmental science

• Business, administration, finance and economics

• Engineering, computer science and mathematics

• Medicine, pharmacology and veterinary science

• Social sciences, arts and humanities

The search was performed on December 7, 2009 and repeated on March 15, 2011 returning in total 788 articles. An article was excluded as soon as it was clear that it did not satisfy the inclusion criteria. Each excluded article was tagged with an exclusion reason. The full text was read by the first author (CÅ) for articles thought to meet the inclusion criteria. In cases of uncertainty, she discussed the inclusion of certain studies with the co-author, JAR.

Since we were not able to search abstracts within Google Scholar, we were concerned that the title-based search may have missed important articles. Two relatively recent and comprehensive literature reviews [8,17] concerning open source software development were retrieved as a part of the Google Scholar search. The references of these reviews were evaluated, and articles found to meet the inclusion criteria (n = 10) that had not previously been identified were included. (Twenty-five literature review references were labeled as duplicates since they had already been identified through the general search.) In the end, 47 articles were included in our systematic review (see Appendix I included as an additional file for a list of the articles).

### Inclusion and exclusion criteria

We included multidisciplinary articles that empirically evaluated open source software development characteristics and barriers. We excluded articles not relevant to our research questions. These included the exclusion of such topics as: determining the success of an open source project (we were most interested that the project had created a product, not how many times the product had been downloaded), comparisons of efficiency between open source and proprietary methods, open source's impact on innovation models, the adoption or use of open source products (again we focused on creation, not use), analyses of how firms profit from open source, among many other topics.

Articles must have been based upon empirically generated evidence, either quantitative, qualitative or mixed methods. Theoretical articles, simulation models and non-academic texts were excluded. Articles were mostly retrieved from peer-reviewed journals or academic conferences, however, four working papers were also included. We decided to include these articles as long as they met the quality criteria. Only articles written in English were included.

### Quality assessment

Each article's quality was evaluated by answering the following questions:

• Is the article based upon empirical evidence?

• Did the article clearly articulate its research question or hypothesis?

• Was the study design clearly articulated indicating how the data was gathered and analyzed?

• Are the findings justified based upon the study design and data?

The quality assessment was kept simple because the articles came from diverse academic disciplines in addition to being a mixture of quantitative and qualitative research. As long as the article managed to rudimentarily answer the quality assessment questions, it was included. Only three articles were excluded for quality reasons (two for study design [18,19] and the other for findings not justified based upon the data [20]).

### Synthesis

Synthesis (the step of compiling the findings of the included studies) proved complicated due to the variety of research methods and academic disciplines. No two quantitative surveys were the same. Different groups were targeted, different questions asked and different theories were used to evaluate the results. Therefore, it was inappropriate to perform a meta-analysis on the quantitative data due to the heterogeneity of the data. The qualitative data provided rich descriptions and better contextual understandings of the open source model. We determined that the most relevant method to synthesize the studies was to focus on the findings or conclusions of the articles, keeping in mind the context in which the conclusions were made.

Treating the findings in this way allowed us to use a qualitative synthesis approach called meta-ethnography. This approach, developed by Noblit and Hare [21], allows researchers to synthesize findings across studies according to the following phases:

1. Getting started: In this phase the researcher chooses the topic for synthesis.

2. Deciding what is relevant to the initial interest: This phase sets out the search strategy.

3. Reading the studies

4. Determining how the studies are related: In this phase the researcher tries to find common and recurring concepts or themes across the included articles.

5. Translating the studies into one another: Here the researcher takes the findings from each article within the context of the article and compares and contrasts them to the next article's findings.

6. Synthesizing translations: Finally the researcher constructs a high-level interpretation from the data.

## Results

### Description of the studies

The forty-seven studies included in the review were published between 2000 and 2011. Research methods used were qualitative (n = 19), quantitative (n = 22) and mixed methods (n = 6). In the quantitative studies sample sizes were both large (2700+ respondents [22]) and small (13 respondents [23]). The most common qualitative methodology used was case study. Academic disciplines represented are economics, information systems, law, management and social sciences. Two-thirds of the studies were focused on large, active open source software development projects meaning that the findings are skewed in favor of successful projects with an active developer base (which are actually the minority of open source software development projects) [24-27]. When presenting the results, we tried to emphasize where this bias is present. The principle findings for each of the studies are summarized in a table which is available upon request.

### Synthesis results

Table 1 summarizes our synthesis results. These are characteristics repeatedly occurring in the research that we interpret as pertinent for applying open source concepts to drug discovery. The categories emerged from the articles and give a good representation of major findings. Each characteristic is described in greater detail within this section.

**Table 1:** Open source software development characteristics

Category	Characteristic
Attracting participation	• Motivations are diverse; no one singular motivation dominates for individuals. • Sustained participation of contributors is difficult to achieve but can be influenced by stressing the importance of the individual's contribution as well as fostering an atmosphere of learning. • Firms are profit-seeking and, as to be expected, motivated by economics. • Individual and corporate motives can co-exist harmoniously.

Management of volunteers	• Contributors are not assigned to tasks; they choose the tasks that suit them. • Contributors complete tasks at their leisure and have freedom of design. • Decision-making is consensual in large projects. • Successful project leadership for large projects follows a motivational style.

Control mechanisms	• Large projects are controlled by small groups of core members. • The quality of the changes is controlled through the peer-review process. • Modular designs allow for incremental and expedient growth, as well as speedy retraction of faulty modules. • To attempt to control the volume of information circulating in the community, rules and norms are communicated and expected to be followed. • Newcomers to large projects tend to enter through an informal but controlled introduction.

Legal framework	• Large projects take measures similar to corporations to protect their work. • Contributions are copyrighted with due credit given. • Most contributors adopt a license that is known and trusted. • The choice of license is not necessarily as important as the norms that contributors follow.

Physical constraints	• The end-product is intangible, non-rival with a marginal cost near zero. • Contributors must have access to a minimum technical infrastructure.

#### Common misconceptions

Firstly, it is important to dispel some common misconceptions. Open source is often portrayed in the media as a way to get an army of programmers to volunteer their services to jointly develop bug-free software in record time. Linux and Apache are the most commonly mentioned, however, the most exceptional. In reality, the vast majority of open source projects has only one or two developers and has not yet released any operational software [17,24-27]. In most projects the project leader is the project founder and the project maintainer [27]. There is little collaboration, and most development actually takes place in isolation [22,28].

Open source projects are not all community-based. They may also be initiated and controlled by a firm. Mozilla Corporation decides who can change the official version of the popular, open source web browser, Firefox. Anyone can download the source code and modify it, but they cannot call the new version Firefox unless the changes are performed through Mozilla's official change control process. It is the firm's challenge to attract external developers to a firm-led project [29]. Mozilla tries to develop a sense of ownership and excitement about the software which will lead to contributions [30]. Many firms also pay employees to contribute to both firm-led and community-led projects, such as IBM's involvement in the community-driven Apache. Firms save money not only by using open source software but also by receiving testing and improvement feedback gratis from volunteers [31]. Firms are an active player in the open source movement [32]. According to one comprehensive survey in 2003 more than a quarter of open source software developers were paid by a commercial firm to make contributions to open source projects [27].

A final misconception is that all programmers involved in open source are selflessly and altruistically donating large amounts of time to code and bug-fix. True, many programmers contribute large amounts of time. Surveys have found that developers expend on average 11 hours a week on open source efforts (a median of 7 hours), more than 25% of a standard work week [27,33]. It is important to examine these figures, though, as they lump together many different types of developers. As we will discuss later, it is common in large projects to have a small, core team of members who dedicate significantly both in time and output. Additionally those programmers employed full-time to contribute will have above-average participation levels. Therefore, these two groups skew the averages. The majority of programmers participate only occasionally, and their contributions may take little or no effort.

Lakhani and von Hippel found that participants of an Apache help forum used on average 1-5 minutes to answer a question [34]. They were able to provide assistance so quickly because they only answered questions where they already knew the answer and ignored those they did not. The authors called answering questions "a costless side-effect" to the main objective of learning about potential problems. In another similar example, newcomers to Freenet would often donate previously developed code as a feature gift [35]. Since little to no modifications were necessary, the cost of sharing the code was near zero yet the benefit to the community was significant.

A review of the motivations (detailed below) shows that very few participants are actually selfless. Common motivations reported are those where the programmer satisfies a need - learning, using the code for his/her own purposes, monetary rewards or demonstrating his/her ability to assist in finding a job or being promoted. Research has demonstrated that the amount of effort expended is correlated to the programmer's selfish motives [36-38]. Ghosh found in a comprehensive survey that the majority of contributors classify their relationship with the community as "I take more than I give" [22].

#### Attracting participation

Attracting participation is a prerequisite for collaboration and likely the most differentiating factor between an individual's hobby project and a successful open source project [28,32,39]. Schweik et al. argues that attracting contributors determines the quality, scalability, longevity and ultimately the success of a project [32]. Projects with higher activity levels are in more advanced states of development and more likely to attract developers [40,41].

Attracting participation presents the project founder with the challenge of attracting highly skilled programmers in scarce supply. A programmer needs to understand a project's architecture, programming language and standards before writing a single line of code [35,42]. Without these skills, postings will appear to be written in a foreign language. Not only is the supply scarce, but the demand is great with hundreds of thousands of open source projects competing for programmers' abilities.

Motivations for participating are diverse; no one singular motivation dominates for individuals [22,36,43]. The most commonly reported individual motivations are displayed in Table 2. Benbya and Belbaly found that economic, social and psychological motives can coincide [44].

**Table 2:** Individual motivations for contributing

Motivation	Description	Reference
Economic	The programmer earns money from his/her contributions.	[22,27,31,37,38,43,45,46]

Enjoyment	The programmer likes contributing. It is fun.	[33,34,38,43,45-48]

Identity	The programmer identifies him/herself as an open source programmer and wants to maintain that identity.	[33,45,49]

Learning	The programmer wants to learn about the software, programming language, architecture, existing problems, new features, etc.	[22,33-36,43-45,48,50-52]

Networking	The programmer wants to develop a peer network.	[27,36,43]

Own use	The programmer needs the code for his/her software.	[27,33,35,38,43,45-48,50,51,53]

Political	The programmer believes that "all software should be free".	[27,33,34,39,43-45,54]

Signaling	The programmer wants to signal (or demonstrate) his/her skills to a wider audience, possibly to assist in finding a job, being promoted or another extrinsic reason.	[34,35,38,45-48,50]

Sustaining participation is difficult to achieve. One study reported that large-project contributors leave a project within one year [47]. Another study focusing on both large and small projects found that the median length of project participation was 1.2 years [27]. Fang and Neufeld state that "80% of open source software projects fade away due to insufficient long-term participation" [51]. They found in one case that motives changed over time, and those programmers that continued to learn and develop from their role in the community as well as those that identified highly with the community were much more likely to continue participating [49,51]. This finding was reinforced by other studies that found that individuals who feel that their contributions are highly valuable for the project are likely to become more engaged [27,30,38,45]. Those programmers who participate because they need the software for their own use typically exit the community when their needs are met [47]. Since many participants will only participate marginally, a project needs to attract many more participants than it actually needs [55]. Attracting participation and then sustaining it presents one of the biggest barriers to keeping a project moving forward.

Due to the difficulty sustaining participation from individuals, it is often desirous to attract firms to a project. They offer considerable and stable resources. As mentioned earlier, being paid to contribute is evidenced in above-average participation levels [33,46]. When firms actively contribute to a project, they contribute more than half of the code [29]. They also perform necessary non-development activities such as testing and writing documentation [29]. Firms are profit-seeking and, as to be expected, motivated by economics [50,56]. Firms contribute either because they sell complementary products to open source software (like hardware or consulting) or they use the software and need to specialize it for their own needs [50,56,57].

Individual and corporate motives can co-exist harmoniously. Approximately one-third of the projects hosted on SourceForge (this includes both firm-led and community-led) receive contributions from both groups [29]. In order to achieve this harmony Bonaccorsi and Rossi assert that firms have to conform to the values of the community [56]. If the programmers do not trust the firm, they will not contribute.

#### Management of volunteers

Managing volunteers is a tricky business. As volunteers they can easily quit volunteering if they become disgruntled or disillusioned with a project. Even worse in open source, they can take the code and create a "fork" (splitting the project into two or more projects developed separately), dividing the original project's valuable resources as fellow programmers join the new fork [30,58-60].

This loose affiliation does not suit standard project management practices where leadership assigns tasks and deadlines. Rather successful project leadership for large, open source projects follows a motivational style. The role of the leader in large projects is to motivate the community, keep the project moving forward towards a common vision and attract more developers [23,38,61]. To do this, a leader needs to be a good programmer with excellent knowledge of the project, but also be a trusted member of the team aligned with the objectives of the community [38,61].

To ensure that decisions are aligned with the community's objectives, decision-making is a transparent, consensual process. Discussions leading to decisions are held publicly, allowing anyone to join the discussion. The resulting decisions will be consensual with leaders being especially attentive that any criticisms are addressed. This is true in both community and firm-led projects. Episodes where this model is not followed can lead to conflict within the community [59].

Keeping the project moving forward is a challenge when contributors choose to perform only the tasks that suit them. Sometimes they choose from "To Do" lists which provide rough, high-level requirements [61,62]. More often a developer will perform a task without initially alerting the community [24,62]. Bugs are reported with the fix attached [62,63]. This ability to perform work without making a public commitment is important. Less-skilled individuals have the opportunity to attempt to solve a problem or create a feature without fear of public failure. If they fail, no one will know because they have not publicly committed themselves. When they succeed, the code is made available [61].

In addition to a lack of resource planning, there is also no evidence of project planning (scheduling, listing deliverables, etc.) [17,24,63]. Deadlines are virtually unheard of within open source projects [47]. Many projects experience a slow pace of development with few or no changes made during a year [26]. Programmers contribute according to time and interest and have freedom of design. Contributors determine themselves how they want to code a solution to a task or bug [61,63].

#### Control mechanisms

In order to develop reputable, high quality software through volunteers, a certain level of control is necessary. Control is maintained through a variety of ways including tight restrictions over who can change the official source code, peer review for quality control, and enforcement of community norms and rules.

Large projects typically have an organizational structure of three concentric circles:

• An inner core team made up of a handful of individuals who are responsible for most of the output including the maintenance tasks [28,35,64,65]. These individuals have "committer" status meaning that they are the only ones that can update the official code and release it. This allows the community to ensure that new code is properly tested before being released as an official version. It also ensures that rogue elements do not release malicious code. In firm-led cases, committer status may be restricted to firm employees [42]. Finding individuals who will act as a committer and perform many of the mundane projects tasks is a barrier to maintaining project momentum.

• A middle circle made up of (hopefully) large numbers of developers who generate code, perform peer review and fix bugs [64]. It is likely that a large number of these individuals will be inactive [51].

• The outer circle is comprised of (again hopefully) many individuals who report bugs but do not code [64].

Before a committer releases a new or modified piece of code, it must have successfully met the community's quality control criteria. This is typically performed through the peer-review process [62]. The new code is made available to the community for review and test. On many of the larger projects, code will not be included in a release until a set number of developers have reviewed the code [64]. Since peer-review is a public activity (archived in the publicly available mailing lists or discussion groups) it is also a useful learning tool where potential community members can become familiar with the code and learn from the mistakes of others [64].

If it is discovered that there are problems with a version of the software, the modular design typical of open source projects allows for quick review, fix or retraction. Modularity allows contributions to be made through small, independent tasks that can be easily integrated according to the project's standards. Modularity has several advantages: new modules can be quickly written, a programmer does not need to understand the entire application to code a module, and modules can be quickly retracted if necessary [24,60,66].

Large projects with hundreds of members can be rapidly swamped by postings from members and outsiders, making it difficult for members to process and react to all of the information. In an attempt to control the volume of information, rules are communicated and expected to be followed [45]. For example, it is often required to consult frequently asked questions and the list of known bugs before posting a bug report [34,61].

Unwritten norms control the behavior of the community. Community members who defy the norms and stated rules risk being "flamed" (publicly, and often harshly, admonished). Stewart and Gosain [54] define the norms as:

• "Do not fork,

• Do not distribute the code changes without going through the proper channels,

• Always give credit; never remove someone's name from a project without that person's consent."

The risk of being flamed, mocked or simply ignored makes newcomers careful about their introduction to the community. They tend to enter through a controlled, unofficial introduction, called a joining script. This typically starts with a period of "lurking" or observing the project unannounced. Once a comfort level is achieved, they move to the next level reporting bugs, possibly followed by developing code and with sufficient commitment and demonstrated ability becoming a committer [35,42]. Becoming a committer generally entails a demonstration of project competence, knowledge and commitment. To become a committer is to be recognized by the community as one of its best developers [38,63,67].

#### Legal framework

Open source does not operate in the absence of a legal framework. Rather it capitalizes on derivations of common legal practices. O'Mahony demonstrates that large, open source projects take measures similar to corporations to protect the reputation of their products, oftentimes creating non-profit legal entities. These legal entities allow projects to protect volunteers from liability and establish trademarks. Where as anyone is allowed to extract the source code, change it and re-release it, if the project is trademarked they are not allowed to do so under the same brand name. Trademarks legally prevent the release of unauthorized versions that may be of inferior quality or divergent from the strategic aims of the project [68].

Contributions are copyrighted with due credit given. Observers often question why programmers are willing to give their work away for free. O'Mahony responds that one needs to "examine what is given away (code) and what is retained (rights)" [68]. Contributors utilizing one of the Open Source Initiative's licenses maintain a level of control regarding how their contributions may be used and ensure that adequate attribution is given. By choosing a viral license (a license that impacts future versions), contributors have legally bound all future versions to the same licensing terms.

Most contributors adopt a license that is known and trusted. The viral license, GPL, is the most popular license amongst open source software development projects [25,26]. One theory for this is that volunteer developers shy away from unfamiliar and distrusted licenses [39]. GPL was created by Richard Stallman and is used by Linux, giving programmers the sense of security that they desire. This need for familiarity may explain why software is simply not placed in the public domain, avoiding the entire licensing dilemma. A programmer could do so by simply declaring that the work resides in the public domain. As Maurer and Scotchmer state, "this strategy would be simpler to implement than the elaborate licenses that open source actually uses" [9].

With the involvement of more and more proprietary software firms in open source communities, the viral GPL licenses has become a barrier. Viral licensing poses significant risks to these firms with the potential for inadvertently mixing their proprietary software with the viral GPL-software. Belenzon and Schankerman found that programmers strongly sort by license type, meaning that developers consistently use either viral or non-viral licenses. Those that consistently use non-viral licenses are more likely to contribute to projects sponsored by corporations [25]. This may explain as of 2008 why non-GPL projects on SourceForge receive more contributions than GPL-based projects [25]. Vetter argues that the viral terms impede deployment, slow its adoption rate and inhibit interoperability and compatibility [69].

The choice of license is not necessarily as important as the community's norms. Projects licensed under non-viral licenses (such as Apache web server) can be legally copied and sold as a proprietary product (under a different name). Where as this may appear superficially to be a money-making opportunity, in reality the potential rewards are small. Firstly, because the savvy programmer will know that the software is available open source and will not pay for a similar proprietary product. Secondly, because it violates all of the norms mentioned earlier, this new proprietary version will not benefit from the future improvements of the open source community. Programmers will continue contributing to the open source version [68,69].

#### Physical constraints

While we have addressed many structural and legal characteristics, it is important to mention the actual end-product of open source software development - the software. It is intangible, unless burnt to a CD or placed on a memory stick. It is what economists call non-rival. An apple is rival. If one person eats it, no one else can. Software is non-rival. Many people can make copies of it and use it without impacting each other. Software also has a marginal cost near zero - it costs almost nothing to produce one additional unit. This means that if the development labor costs are free, it has ultra-low design and distribution costs [24]. Other examples of non-rival goods are knowledge and broadcasted television.

In order to contribute to an open source project, programmers must have access to a minimum technical infrastructure. All open source projects must be hosted on a website that is accessible to the general public. This requires the creation and maintenance of a website and server. Individual programmers must possess common communication tools (a computer, e-mail, file transfer and access to discussion groups) as well as have relatively fast and efficient access to the Internet [45]. Bonaccorsi and Rossi attribute the preponderance of European and American programmers to the superior Internet connectivity available in these areas [56]. These technical requirements are a barrier to entry.

## Discussion

The final phase of the meta-ethnography approach is to synthesize the results, constructing a high-level interpretation from the data. We have taken the characteristics pertaining to open source software development and applied them to drug discovery.

### Limitations

Before discussing the identified characteristics' applicability to other settings, we must be clear about the limitations of our systematic review. We have attempted to be transparent and unbiased about each step performed to come to our summary of findings. Unfortunately though, it is impossible to reduce all phases into mechanistic, reproducible procedures. Our subjectivity has influenced our assessment.

The relatively large number (n = 10) of additional papers identified through the literature reviews demonstrates the difficulty of performing a multi-disciplinary review. Few databases support this breadth with Google Scholar being the most comprehensive. However, with its limited search capabilities, important papers may have been missed.

The full text articles were read by only one of the authors. Articles uncertain to have met the inclusion criteria were discussed. Therefore, theoretically more or fewer studies may have been included if the full text articles had been read by multiple individuals. We attempted to eliminate this bias by defining stringent inclusion and exclusion criteria, and when in doubt we included the article.

Choosing which characteristics to portray was also subjective. We consciously attempted to choose those characteristics that reappeared in multiple studies as well as those originating from high-quality articles while being wary to the bias that the bulk of the research focused on large, successful projects.

When translating relevant characteristics from software to drug discovery, we present our views of the possible applicability. This is not based on a given methodology but is our interpretation.

Despite these methodological weaknesses, we believe that we have offered a useful starting point for taking the lessons learned from open source software development research beyond computer software.

### Open source drug discovery

Open source offers exciting prospects for innovation, but can methods used to create intangible software be extrapolated to produce tangible medicines? Firstly, for clarity, it is important to define open source drug discovery. There are many articles [70-72] and books [73,74] proposing and discussing the topic, and thereby several interpretations. We will use the definition that we believe is the most exact from a recent summary report by Results for Development Institute (R4D) [75]. R4D defines three types of "open" when examining open source in the context of drug discovery:

**• Open access: **Free and open access to research data

**• Open collaboration: **A collaborative workflow across organizational boundaries

**• Open rules: **The rules that mandate the openness (for example, contracts, intellectual property and licenses)

To be unequivocally "open" projects must adhere to all three requirements.

Notice that R4D's definition does not state where within the phases of drug discovery and development these three "open" types are applied. This is an area of debate and uncertainty. There seems to be a general agreement that open source is a viable model for precompetitive activities [75]. Precompetitive within drug discovery is generally considered to be all stages prior to patenting a promising, optimized lead compound [76]. Applying open source beyond this point may only make sense for drugs targeted at "neglected" diseases, those diseases largely ignored by industry since the market is considered unprofitable. Medicines developed for these diseases are primarily done so through product development partnerships, organizations that focus on developing new medicines and diagnostics for diseases inherent to low and middle income countries. These medicines may or may not be patented. Since it can be argued that the drug development phases (not only the discovery phases) for neglected diseases are non-competitive due to the absence of a profitable market, open source may be relevant for preclinical testing and process development. The applicability of open source to clinical trials is questionable since open collaboration is not appropriate for rigidly designed and tightly controlled trials and open access not applicable for confidential patient data. However, one may argue for a hybrid approach to clinical trials where all involved use a semi-open solution with information shared through a closed extranet.

For the purposes of our examination we will confine our analysis to drug discovery (all phases prior to preclinical testing), although we believe that it would be useful in another study with a broader analysis to examine the applicability within drug development for neglected diseases. Below we contrast the open source software characteristics identified through this review to drug discovery.

### Attracting participation to drug discovery projects

The research for open source software development has demonstrated that attracting participation is critical in order to move the project from an individual's hobby project to a successful open source project. Numerous motivations must be present to attract sufficient participation. Are there diverse motivations within drug discovery both for individuals and firms?

We hypothesize that personal motivations for scientists to contribute will not largely differ from those of software programmers. Motivations such as enjoyment, identity, learning, monetary rewards, networking, political and signaling are just as viable for scientists as they are for programmers [71]. The problem is that drug discovery often also requires a laboratory and physical resources, and these motivations are not sufficient if a monetary outlay is required. Munos divides pharmaceutical research and development into those activities that require "intelligence and intuition, but little infrastructure" against those that require physical assets [72]. These motivations fit well with the former knowledge-based activities (he gives examples of identifying targets, understanding metabolic networks, and designing computerized disease models). However, if open source drug discovery was limited to the knowledge-based tasks, no new drugs would be discovered. Therefore, either firms must also be motivated to participate, providing access to laboratories and physical supplies, or projects must receive funding through research grants (or both).

This latter option is not unlike firm-led open source software development projects where a single funder employs programmers to work on a project and maintains control over the source code. Single payer open source drug discovery projects can also abide by the definitions of open access, collaboration and rules. We offer in Table 3 potential motivations for both private and public organizations to participate in and/or fund open source drug discovery tasks. These are not motivations for an organization to sponsor the entire drug discovery process, but only to participate in or fund discrete, time-limited tasks such as the curation of genetic disease data, validation of the feasibility of assays and models on established targets, or sharing of data related to the identified compound. In line with the finding that firms are motivated by economic returns, many of these motivations are based upon the assumption that they will lead to financial rewards. For example, one potential motivation for an emerging country pharmaceutical manufacturer is to position itself for the role of manufacturer.

**Table 3:** Potential motivations for organizations to participate in an open source drug discovery project

Innovator	Funder or Performer?	Motivation
Emerging country biotechnology, pharmaceutical or vaccine manufacturers	Both	• Employee retention - allowing employees to participate in external projects as a percent of their work week to increase work satisfaction • Employee training - new employees can receive on-the-job training by contributing to open source projects and receive performance appraisal by the community • Monetary - projects with external funds may hire industry to perform specific tasks • Monetary - payment for the drugs manufactured • Open innovation - receive ideas and feedback from external sources in exchange for assurances that the end result will be made available affordably to developing nations • Simplify regulatory process - making the research data publicly-available and open to scrutiny may engender more trust on behalf of the regulatory body

Large, multi-national biotechnology, pharmaceutical or vaccine manufacturers	Both	All from above plus: • Reputation - corporate social responsibility • Priority regulatory review - if they champion a project for a neglected disease, they may receive a priority review voucher (open source may speed up the process to the voucher)

Small, niche biotechnology or pharmaceutical manufacturers	Both	• Monetary - may open external funding opportunities that were otherwise closed • Monetary - payment for the drugs manufactured • Open innovation - receive ideas and feedback from external sources in exchange for assurances that the end result will be made available affordably to developing nations

Charities	Funder	• Aid - Developing country scientists and innovators gain free access to utilize the research or further develop it • Efficiency - tasks should be delivered faster and cheaper • Transparency - donors can readily see how their money is being used, rather than relying solely on reporting mechanisms

Governments	Funder	• Aid - Developing country scientists and innovators gain free access to utilize the research or further develop it • Efficiency - tasks should be delivered faster and cheaper • Innovation and subsequent tax revenues - anyone can take the publicly-funded research and further develop it for other aims with potentially profitable outcomes • Transparency - citizens can readily see how their money is being used

Product development partnerships	Funder	• Competition - may create a competitive landscape between individuals and/or organizations attempting to complete the task first • Efficiency - tasks should be delivered faster and cheaper • Transparency - donors can readily see how their money is being used, rather than relying solely on reporting mechanisms

Academic institutions, government research organizations and research hospitals	Performer	• Efficiency - feedback on research can be received long before publishing • Fair play - anyone can take the publicly-funded research and further develop it for other aims • Transparency - citizens can readily see how their money is being used

Contract research organizations	Performer	• Monetary - projects with external funds may hire CROs to perform specific tasks • Signaling - demonstration of abilities for potential employers

Generics manufacturers	Performer	• Monetary - payment for the drugs manufactured

### Management of volunteers in drug discovery projects

Drug discovery, unlike computer programming, follows a more rigid project management process. Tasks must follow a scientifically-prescribed process. It is helpful to duplicate certain tasks (e.g., laboratory experiments) to confirm results but not all as this adds unnecessary expense. Therefore, much of the flexibility allowed programmers is not relevant for drug discovery. As volunteers, scientists will certainly pick the tasks that match their motivations. This means that open source drug discovery projects must have a strong project management approach, continually articulating the discrete tasks required at the time as well as finding the supporting funds. This is an ideal role [72,75] for a Product Development Partnership (PDP), organizations that focus on both drug discovery and development for neglected diseases. Not only are PDPs familiar with the science behind the diseases, they are also experts in developing new medicines relevant for their target markets (inexpensive, durable and straightforward). They may also play a role in launching the product locally [77].

Munos states that PDPs have already "adapted the open-source concept and combined it with outsourcing to create a new, low-cost business model...in which a part of R&D is open-sourced while the rest is outsourced. To function, however, it needs strong project leadership and expertise in the minutia of drug R&D..." [72]. He sites the Medicines for Malaria Venture which gathers its projects through open calls, uses a Scientific Advisory Committee to tightly manage its projects and outsources tasks to collaborative research teams.

Other candidates to fulfill the project management role may be disease-specific charities and governmental research organizations. Regardless of who performs the role of project manager, a significant challenge, similar to software development, will be to keep the project moving forward when contributors choose to perform only the tasks that suit them. We have already established the need for external funding in open source drug discovery. This funding will undoubtedly be linked to project milestones with associated delivery dates. We argue that some funding will likely need to be used to pay employees (or others involved) to work on the project. This will give the project necessary momentum, without which volunteers may become disgruntled and disinterested. Again this does not deviate from many firm-led open source software projects that pay employees to code the software.

### Control mechanisms of drug discovery projects

There are two central questions regarding control mechanisms that will determine the applicability of open source to drug discovery. Is drug discovery modular, and how will the quality be managed?

Drug discovery for novel products may be broken down into four phases, each taking years to complete:

**1) Basic Research **focuses on gathering detailed knowledge of the disease organism and how it interacts with the human body [78]. This phase is performed mainly by academics and public-sector researchers through grant funding.

**2) Target Identification and Validation **is the process of determining biological or chemical targets which may interfere with the disease organism [78]. This phase may be further broken down into target screening, validation and early assay development [79]. It is performed by biologists, biochemists, geneticists and bioinformaticians employed principally by governments or universities [78].

**3) Lead Identification **is the process of identifying compounds that have desirable effects on the validated targets [78]. Tools used to identify leads are high throughput chemistry, combinatorial chemistry, computational biology as well as literature searches of known compounds. Industry is typically best at high throughput chemistry as they own the proprietary chemical libraries necessary. The other tools may be performed either in universities, government research facilities or industry. However, industry medicinal chemists, due to their optimization and development experience, are more apt to identify lead compounds that will succeed in optimization (meeting, for example, safety and absorption requirements).

**4) Lead Optimization **focuses on modifying a handful of compounds for in vivo results such as bioavailability and the avoidance of toxicity [79]. The compound that is successfully optimized will start the development phase including scaling up production quantities as well as animal safety studies.

Each of these phases can be further broken down into many discrete tasks demonstrating that drug discovery is modular. With project management constantly identifying the tasks currently needed, scientists should be able to find small, discrete tasks that motivate them to contribute. Yet there is no clear formula how to subdivide these processes into concrete tasks. Maurer provides example tasks for Lead Identification [71]; volunteers contribute by searching online databases for known leads against a specified target, running computational chemistry simulations, and performing physical chemistry experiments to verify the simulation results. Those lead compounds predicted to be promising by multiple volunteers would move onto optimization.

The project manager also needs to ensure that multiple external contributors are generating output of a consistent quality. Many questions regarding the quality of external contributors can arise such as the quality of the compounds used for screening, inter-lab equipment differences, accuracy of data extraction, etc. [80]. This incremental quality is monitored through publishing in peer-reviewed journals and as well as by the use of Scientific Advisory Committees. A recent experience of an open source drug discovery project demonstrates that perceptions of what constitutes peer review within open source can differ [81]. Some believe that publishing results for all to see on a publicly accessible website is sufficient (similar to open source software development), while others insist that results must be published in a peer-reviewed journal both to secure quality control and wider circulation of the results. With a dearth of ongoing open source projects, quality control will likely need to remain in the traditional realm of peer-reviewed journals until vastly more scientists begin to participate in open source projects.

### Legal framework of drug discovery projects

As with software, legalities need to be simple, understandable and trusted. They become more complicated, however, because the principal legal protection used for drug discovery is patents, not copyright. Where as copyright protects an original work (such as a document, song or painting), patents protect ideas. Software code is considered an original work and, therefore, automatically covered by copyright. Patents are not automatic and must be sought. Applying open source models to patent-heavy industries presents many challenges.

Designating a product as free to use, modify and distribute is more complicated with patents. The equivalent of an open source (copyright) license for patentable ideas is the use of the public domain. Ideas residing in the public domain are not owned by an individual or corporation--they are the property of the public and, therefore, may be used, augmented and manufactured by anyone without crediting or notifying the innovator. This may be cause for concern for innovators who are interested in maintaining some level of recognition and control over their inventions. In these instances, an alternative exists--the invention may be patented and made available to the public by a license similar to those used by open source software, for example a royalty-free license to a patented technology. This, however, may be impractical as patenting is expensive and, therefore, there is little incentive to do so when the ultimate product (or at least the design) is intended to be free-of-charge.

If licenses are to be used rather than the public domain, one should be critical to the use of viral licenses (those that infect future versions). Firstly, this may be illegal for licenses of patented products. Patents have a much shorter lifespan (typically 20 years) than copyrights (lifetime of the author plus 50-70 years depending upon the country). Once the patent expires, it automatically becomes a part of the public domain where licenses are no longer relevant. Future uses of the patented technology cannot be bound to a viral license after the patent has expired as it resides in the public domain. For products typically protected by copyright, viral licenses can be highly contentious, especially for firms. When choosing a type of license, it is important to carefully weigh the advantages and disadvantages of each license.

### Physical constraints to drug discovery projects

Now back to our original question, can methods used to create intangible software be extrapolated to produce tangible medicines? We have demonstrated that drug discovery may be broken down into both tangible and intangible tasks, with the tangible ones requiring access to expensive facilities and physical goods. The intangible tasks of knowledge creation are similar to software development in that they may be performed and communicated virtually. This knowledge, however, may be either rival or non-rival depending upon the drug discovery phase. Precompetitive data may be used by all with the impact of improving the pace and quality of multiple drug discovery projects [76]. However, competitive (i.e., patented) knowledge is rival, only available to the innovator or licensee.

The marginal cost of a new drug does not come close to zero until the medicine is being manufactured. The cost to manufacture the first pill (including all research and development costs) is exceedingly high. It is the second and subsequent pills that cost little to produce.

These characteristics are contrary to the physical characteristics of software. The question is whether these physical constraints can be overcome by modifying the model to include funding and paid project leadership to guide volunteers (as well as paid employees) through the drug discovery phases.

## Conclusions

We have attempted to provide an understanding of open source software development characteristics for researchers, business leaders and government officials who may be interested in utilizing open source in other contexts, specifically within drug discovery. We have done so by examining the existing research through a systematic review and extracting characteristics common to open source software development that we believe are relevant when building an open source drug discovery initiative.

Open source is a desirable model for drug discovery because it offers the potential benefits of research being performed quicker with reduced labor costs while avoiding duplication of efforts. It is particularly relevant for neglected diseases where insufficient incentives exist to promote private investment. New drugs for these diseases are discovered and developed primarily with the use of public or philanthropic funds. From a funder's perspective, there are few downsides in grantees adopting an open source approach, ensuring transparency in the use of funds and potentially speeding up the project through supplementary free labor.

Can a new pharmaceutical be developed entirely through an open source model? Likely not. However, a new drug for a neglected disease may be shepherded up to clinical trials utilizing a hybrid open source model combining open source with other development models such as fee-for-service outsourcing. To assist with this development, we believe that further research is needed on business modeling, incentive development and the impact of the use of the public domain. It is important that this research includes expert input from researchers, the pharmaceutical industry and PDPs to assess the practicality and relevance of open source drug discovery at a task level.

## List of abbreviations

GPL: General Public License; a copyleft license, giving anyone the freedom to use, modify and distribute software with the caveat that all modifications must also adhere to the GPL; PDP: Product Development Partnership; organizations that focus on developing new medicines and diagnostics for diseases inherent to low and middle income countries; R4D: Results for Development Institute (see reference 75).

## Competing interests

The authors declare that they have no competing interests.

## Authors' contributions

The review was designed by CÅ and JAR. CÅ performed the review and wrote the first draft. Subsequent drafts were revised by all authors.

## Funding

This review was funded by the Norwegian Research Council. They did not play any role in the production of this review or in the decision to submit the manuscript for publication.

## Supplementary Material

Additional file 1 **Appendix I: Articles included in the synthesis**. This additional table references all articles included in the synthesis of the systematic review. Click here for file
