# Supplementary material for: Common characteristics of open source software development and applicability for drug discovery: a systematic review
Source: Health Res Policy Syst. 2011 Sep 28;9:36. doi: 10.1186/1478-4505-9-36 (PMC3206459; doi:10.1186/1478-4505-9-36)
Supplement: Additional file 1 — Appendix I: Articles included in the synthesis. This additional table references all articles included in the synthesis of the systematic review. [file 1478-4505-9-36-S1.PDF]

Appendix I: Articles included in the synthesis

Author(s) (Date)	Type	Software Focus	Reference
Belenzon and Schankerman (2008)	Quantitative	SourceForge	[25]
Benbya and Belbaly (2010)	Quantitative	SourceForge	[44]
Bonaccorsi and Rossi (2006)	Quantitative	Italian firm-based solutions	[56]
Capiluppi, Lago and Morisio (2003)	Quantitative	Freshmeat	[26]
Capra et al. (2009)	Quantitative	SourceForge	[29]
Chengalur-Smith, Sidorova and Daniel (2010)	Quantitative	SourceForge	[40]
Colazo and Fang (2009)	Qualitative	SourceForge	[39]
Crowston and Scozzi (2002)	Quantitative	SourceForge	[41]
Crowston et al. (2007)	Qualitative	Gaim, eGroupware and Compiere ERP	[62]
David and Shapiro (2008)	Quantitative	Diverse	[43]
David, Waterman and Arora (2003)	Quantitative	Diverse	[27]
Fang and Neufeld (2009)	Qualitative	phpMyAdmin	[51]
Ghosh (2005)	Quantitative	Diverse	[22]
Giuri, Rullani and Torrisi (2008)	Mixed	SourceForge	[23]
Hagen (2011)	Quantitative	Qt framework, KDElibs, Asterisk PBX and FreePBX	[31]
Hars and Ou (2002)	Quantitative	Diverse	[36]
Haruvy, Wu and Chakravarty (2005)	Quantitative	Diverse	[37]
Heckman et al. (2007)	Qualitative	Compiere, WebERP, Apache OFBiz, Gaim, aMSN and Fire	[58]
Hertel, Niedner and Herrmann (2003)	Quantitative	Linux kernel	[45]
Howison (2009)	Mixed	BibDesk, Fire and Gaim	[24]
Iansiti and	Quantitative	Open source projects identified in	[57]

Author(s) (Date)	Type	Software Focus	Reference
Richards (2007)		Wikipedia	
Jensen and Scacchi (2005)	Qualitative	NetBeans	[59]
Koch and Schneider (2002)	Quantitative	GNOME	[28]
Lakhani and von Hippel (2003)	Mixed	Apache Usenet help forum, CIWS-U	[34]
Lakhani and Wolf (2005)	Quantitative	SourceForge	[33]
Lee and Cole (2003)	Qualitative	Linux kernel	[64]
Lerner and Tirole (2002)	Qualitative	Apache, Linux, Perl and Sendmail	[38]
Mendonca and Sutton (2008)	Qualitative	Mozilla	[30]
Mockus, Fielding and Herbsleb (2002)	Qualitative	Apache and Mozilla	[63]
Monteiro et al. (2004)	Mixed	Gentoo Linux	[67]
O'Mahony (2003)	Qualitative	GNU Project, Linux kernel, Apache webserver, Debian Linux Distribution, GNOME GUI Desktop and Linux Standards Base	[68]
Roberts, Hann and Slaughter (2006)	Quantitative	Apache	[46]
Rullani (2007)	Quantitative	SourceForge	[55]
Sarma, Lambermont and Clark (2009)	Qualitative	GNOME	[60]
Schweik, English and Haire (2008)	Quantitative	SourceForge	[32]
Shah (2006)	Qualitative	One unspecified open source community	[47]
Shibuya and Tamai (2009)	Mixed	MySQL, OpenOffice.org and GNOME	[42]
Spaeth et al. (2008)	Qualitative	Freenet	[52]
Stewart and Gosain (2006)	Mixed	SourceForge	[54]
Tuomi (2001)	Qualitative	Linux	[66]
Vetter (2004)	Qualitative	Open source licenses	[69]
von Hippel (2001)	Qualitative	Apache	[53]
von Krogh, Spaeth and	Qualitative	Freenet	[35]

Author(s) (Date)	Type	Software Focus	Reference
Lakhani (2003)			
West and Gallagher (2006)	Qualitative	Unspecified	[50]
Wu, Gerlach and Young (2007)	Quantitative	SourceForge, Debian and OpenWebMail	[48]
Xu and Jones (2010)	Quantitative	SourceForge	[49]
Yamauchi et al. (2000)	Qualitative	FreeBSD and GNU GCC Project	[61]
